# Effects of ten weeks dynamic or isometric core training on climbing performance among highly trained climbers

**DOI:** 10.1371/journal.pone.0203766

**Published:** 2018-10-10

**Authors:** Atle Hole Saeterbakken, Einar Loken, Suzanne Scott, Espen Hermans, Vegard Albert Vereide, Vidar Andersen

**Affiliations:** 1 Western Norway University, Faculty of Education, Arts and Sports, Department of Sport, Food and Natural Sciences, Campus Sogndal, Sogndal, Norway; 2 University of Exeter, Department of Sport and Health Sciences, Exeter, United Kingdom; University of Illinois at Urbana-Champaign, UNITED STATES

## Abstract

This is the first study to compare the effects of isometric vs. dynamic core training and characterize core-training adaptations using climbing-specific performance and core strength tests in elite climbers. The aim of the study was to compare the effects of attending a progressive core-training program on climbing performance. 19 advanced and elite climbers (7.3±5.6 years climbing experience, red point skill grade 19 IRCRA) were randomized into a dynamic (DCT) or isometric (ICT) core training group and trained twice weekly for ten weeks. The climbers were tested using two climbing-specific core tests (body lock-off and body-lift) and four non-specific core strength tests—one dynamic (superman) and three isometric (trunk flexion and trunk rotation left and right). Between group comparisons showed no differences between the groups at post-test (*p* = 0.328–0.824) and neither group demonstrated greater improvement compared with the other (*p* = 0.300–0.926). The ICT group demonstrated 10.8% and 29.6% improvement in trunk flexion and body-lift (*p* = 0.029–0.037 with no improvement in body lock-off and rotation (*p* = 0.101–0.343). The DCT group demonstrated 5.0–14.9% improvement in the core strength tests (*p* = 0.012–0.043), a non-significant 33.8% improvement in body-lift (*p* = 0.100) and no improvement in body lock-off (*p* = 0.943). In conclusion, none of the training groups demonstrated greater improvement than the other and both dynamic and isometric core training improved climbing-specific test performance. Dynamic training was slightly more favorable although not significantly superior to isometric core training in improving core strength.

## Introduction

In indoor climbing competition, a route consists of 20–50 holds and takes 2–7 minutes to complete at intensities ranging from 71–91% of maximum heart rate [1–3]. In recent decades, climbing performance measured by the difficulty rating of routes, has greatly improved in Sport Climbing World Cup series. However, the training used to improve and develop climbing performance mostly draws on experience-based knowledge, cross-sectional studies [4, 5] and a few intervention studies conducted in climbing [6, 7]

Several cross-sectional studies have reported strength and/or endurance in the fingers, body composition and relative upper-body strength as the most important physical factors predicting climbing performance [4, 5]. Several factors have been investigated as predictors of climbing performance. The most frequently used tests (Bent-arm hang, Dead-hang and finger strength) relate to climbing-specific actions, and the relative contribution of anthropometric factors (height, weight, body fat) and climbing experience and flexibility have also been examined. Between 0.3–97% of climbing performance has been explained by these factors, singly or in combination, depending on climbing performance level and number of factors included in the analyses [1, 4], with duration in a finger grip suspension (‘dead hang’) found to be the best single predictor of performance (r^2^ = 0.66–0.76) [4].

The difficulty level of a route is manipulated by several factors such as grip type (size and shape), distance and position between grips and gradient of the climbing wall. As a result, climbing includes dynamic and often explosive movements with short static positions before the next transition. In elite climbing routes (24–27 IRCRA), the climbers often only have two contact points in the wall (typically, an arm and the opposite foot). To be successful in maintaining positions, reaching climbing holds, clipping the rope to carabiners and moving efficiently, core stability and strength have often been proposed as key factors in elite climbing performance, and although this has never been systematically examined, Muehlbauer and colleagues demonstrated 19–22% increase in core strength (flexion and extension) among sedentary adults after training climbing twice peer week for eight weeks [8]. A strong and stable core may resist rotation of the trunk [9] and allow force generated by feet to transfer to the arms without causing a loss of energy, impairing technique or reducing range of movement of the arms.

Studies have examined core training and performance in running [10, 11], swimming [12], throwing [13], kicking [14] and golf club velocity [15], however conclusive evidence of the contribution of core strength is lacking. [16]. Studies that have demonstrated improvement have utilized progressive core-training programs and included both core strength and stability [13, 17–19]. For example Saeterbakken et al. [13] demonstrated a 4.9% improvement in throwing velocity among handball players after a core-training program. Still, several studies have demonstrated improved core and stability test results without improved performance [11, 12, 20–22].

In the majority of sports, both dynamic and isometric core movements are important [23]. Previous studies into the relationship between core training and performance have utilized isometric (10, 12, 13, 16) or dynamic (9, 11) training protocols singly or in combination (14, 17) with no conclusive evidence of the best approach. However, comparison of the effects of isometric and dynamic core training has not been undertaken and the effects of progressive core training on climbing performance are unknown. Therefore, the aim of this study was to compare dynamic and isometric core training on climbing-specific performance tests in advanced and elite climbers. We hypothesized that test scores in both groups would increase after the intervention, without significant differences resulting for either groups.

## Methods

### Design

The study was a within- and between- groups comparison design. Nineteen climbers were randomized to participate in a ten weeks progressive core-training program consisting of either isometric or dynamic core exercises. All participants were tested pre- and post- intervention using one dynamic and one isometric climbing-specific core test, one dynamic core strength test and three isometric core strength tests. During the intervention, the climbers continued their normal climbing routines.

### Participants

Nineteen climbers (14 males and 6 females) volunteered to participate in the study. The climbers (age 27.4 ± 6.7 years, height 1.74 ± 0.09 m, weight 70.3 ± 10.1 kg) had a mean climbing experience of 7.3 ± 5.6 years. After the pre-test, participants were first stratified (by sex) and than ranked by climbing skills (red-point on-sight climbing performance last year) into pairs (i.e. the two best males were pair one, number 3 and 4 were pair two, etc.). The climbers in each pair were then randomized by drawing into either an isometric core-training program (ICT) or a dynamic core-training program (DCT). Nineteen climbers completed pre- and post- testing (one male elite climber withdrew). There were no differences between groups in climbing experience, climbing skill or the mean anthropometric measurements at pre-test (*p* = 0.552–0.847). For details, see Table 1.

**Table 1:** An overview of the participants.

Group	Sex	Age (years)	Weight (kg)	Height (cm)	Climbing experience (years)	Climbing ability Elite / advance	Climbing performance IRCRA scale*
DCT	Male (n = 6)	26.5 ± 6.2	71.3 ± 7.2	177.3 ± 6.0	6.4 ± 6.6	1 / 5	19.2 ± 2.8
	Female (n = 3)	25.3 ± 3.0	68.2 ± 6.4	169.3 ± 2.1	6.8 ± 2.8	0 / 3	17.3 ± 1.5
ICT	Male (n = 7)	30.3 ± 8.7	77.3 ± 7.3	180.5 ± 6.0	8.6 ± 7.2	1 / 5	22.3 ± 2.1
	Female (n = 3)	24.3 ± 4.1	54.0 ± 1.8	159.7 ± 2.3	3.9 ± 0.7	2 / 1	19.9 ± 4.4

* red-point on-sight last year

The inclusion criteria for participation were minimum one year of climbing experience, free of injuries for the last six months, having a climbing frequency of at least two sessions per week and the climbing skill had to be on an advanced level or better (>15) according to the IRCRA scale [24].

### Ethics statement

The study was approved by the Regional Committees for Medical Health and Research Ethics in Norway (2009/1735/REK Sør-Øst D) and conformed to standards of treatment of human participants in research as outlined in the 5^th^ Declaration of Helsinki. Participants were informed (both in writing and orally) about all testing and training procedures and gave written informed consent to participate prior to entering the study. Participants’ gave their written informed consent (in accordance with PLOS consent guidelines) for their images to be reproduced in this manuscript.

### Testing procedures

Before the pre-test, a pilot study into the test battery was conducted, in which the distance and height difference (i.e. test angle) between hand grip and foot placement in the two climbing-specific core strength tests (body lock-off and body-lift) were examined. With reduced distance or greater height difference between hand and foot placement, grip endurance (rather than core strength) was found to be the limiting factor. Therefore testing procedures were carefully selected to ensure that core strength and not grip endurance was the factor under examination. Core strength tests in neutral spinal and pelvic alignment were also included to test the effect of dynamic and isometric contractions in this position. The exercise known as ‘Superman’ (dynamic version of the prone bridge) has been used in interventions previously [13, 25], but not applied specifically as a test. However, after some pilot testing, we found this exercise to have high ecological validity as a test, providing criteria for correct performance are strictly observed (see methods). Isometric core strength testing involving trunk flexion and rotation has been previously undertaken [8, 25]. After completing pre-testing procedures, eleven climbers (2 females, 9 males, age 28.0 ± 9.0 years, height (1.78 ± 0.60 m and weight 74.9 ± 6.0 kg, climbing experience 7.8 ± 7.3 years) were recruited with an on-sight and red-point indoor climbing skill of 17 (7a French grade) and 20 (7b+ French grade). Participants were tested twice in the body lock-off, body-lift and superman with 3–10 days separating the two sessions (see test procedures). The test-retest reliability (ICC) for the body lock-out, body-lift and superman were 0.790, 0.908 and 0.874, which is consistent with excellent agreement [26].

In both the pilot and the experimental study, the climbers refrained from strenuous training targeting the upper-body (arms, shoulders, fingers and core) 48 hours before testing. Pre- and post-tests were conducted at the same time of the day. The test-order was randomized for each climber. Before starting the test, the climbers performed a non-strenuous 10-minute aerobic warm-up (walking and jogging), 10 minutes of non-demanding traversing of the climbing wall and five boulders (5–10 moves) with medium grips. During testing, 10 minutes recovery between the body-lift and body lock-off tests was permitted and three minutes recovery between the dynamic (Superman) and the isometric core strength tests, with 1–2 minutes recovery between attempts of this tests. Two attempts of the body lock-out and body lift were performed and three attempts of the Superman and isometric core strength tests. Finally, we tested digital strength and endurance in the finger hang test. During testing, participants were verbally encouraged to maximize their performance.

#### Body lock-off

The participant adopted a horizontal position with their preferred foot on a campus rung (40.6 cm long, grip width 2.5 cm., size M, Metolius Climbing, Oregon, US) 20 cm above the ground with the curved edge facing down (S1 Fig). The participant was instructed to press their shoulder blades together (scalular retraction) and lift the other foot to the same height as their foot on the rung without touching it (i.e. unilateral foot support). The participant was told to gripthe campus rung without using their thumbs (S1 Fig) [4], at a self-selected grip width with the little finger within the length of the rung. The distance between the foot and hand rungs was individualized to the height of each climber and the hand support rung was positioned at 50% of the climber`s height. The participant was instructed to lift their body keeping shoulder, pelvis and ankle in a horizontal line. Timing of the test started when the pelvis was elevated from the ground and was stopped when any part of the body touched the ground. Climbers used their own climbing shoes and chalk to improve their grip.

#### Body-lift

The climbers used a shoulder-wide pronated grip to hold a 6 cm fixed beam attached 225 cm above the ground (S2 Fig). A foot chip was placed 185 cm above the ground. The distance between the chip and wooden beam corresponded to the participant’s body length. The climbers were asked to lift their body and place their feet on the chip. Participants had to maintain the foot position for a second (S2 Fig) before lowering the body at a controlled speed to the starting position. The swing of the legs from the lowering phase was stopped completely by grabbing the climbers feet before each repetition. Swinging the legs to gain momentum (kipping the hip) before starting a new repetition was not allowed. Numbers of completed repetitions were used in further analyses. The body-lift test was designed to integrate core muscles with other muscle groups and to resemble climbing. Briefly, to achieve the horizontal position, a dynamic contraction of lower rectus abdominis fibers (pubic tubercle insertion), integrated with inferior external and internal oblique abdominals orientates the lower limbs anteriorly and superiorly. A brief isometric contraction occurs as the foot is steadied on the shelf and, to decelerate the momentum of the lower limbs before a fresh trial is initiated, the core muscles of the anterior trunk and inferior spine and pelvis (iliopsoas) contract eccentrically as the legs are lowered.

#### Superman

The participants adopted a push-up position with their hands on pads on a slide board (Flowin Friction Trainer (Flowin AB, Vintrie, Sweden) with feet against a wall and the pelvis in a neutral tilt. The climbers were asked to slide their arms forward as far as they were able to and return to the starting position while maintaining the alignment (height and tilt inclination) of their pelvis (see S3 Fig). The distance between the feet and the fingertips was measured by placing a pad in front of the two hand pads (see S3 Fig). If the climbers were not able to maintain their pelvic position or failed to return to the starting position, the attempt was not counted. The maximal distance achieved was calculated as a percentage of the climber`s body length.

#### Isometric core strength tests

The tests were performed sagittally (trunk flexion) and with rotation to both sides [20], with the participant’s arms folded across the chest and a non-elastic band attached to a force cell (Ergotest Technology AS, Langesund, Norway) under the arms. Climbers sat on a table with a 90 degree angle at the hip and the feet placed behind the legs of the table (see S4 Fig). The participants were instructed to gradually increase their force output to maximum and maintain the maximum force for five seconds. The highest mean force over a three seconds window was used as identified by the Musclelab software (V10.13, Ergotest Technology AS, Langesund, Norway). The best attempt in each direction used in further analyses.

### Finger hang test

This test measures isometric finger strength and/or endurance and has been applied as a climbing-specific test in several studies which offer more detailed description [1, 4, 6]. Briefly, a participant uses four fingers (the thumb is excluded from the grip) to hang on a 2.5cm Campus rung (medium Campus beam, Metolius Climbing, Oregon, US). To initiate the test the participant is instructed to flex their hips and lift their legs off the floor so that the upper body and trunk hang vertically and the test stops when their fingers lose grip on the beam. Two trials were permitted separated by a 4 minutes rest and the best attempt (i.e. longest duration hang) used in further analyses.

### Training protocol

The isometric and dynamic training programs were performed twice per week for ten weeks. Each session lasted 30–40 minutes and contained four exercises. The training programs were similar in intensity (4–10 repetitions maximum), volume (3–4 sets) and rests between sets and exercises (2 minutes). The exercises were: foot-lift (S5 Fig), arm lock-off (S6 Fig), side-bridge (S7 Fig) and prone-bridge (S8 Fig). The foot-lift and arm lock-off were performed on an artificial bouldering wall. In the foot-lift exercise, the participants hung from two hand grips on a pending wall and lifted one knee or foot towards a foot stance (S5 Fig). In the arm lock-off exercise, participants used their left arm and foot or right arm and foot to maintain a position in the wall and with the free arm reached horizontally as far as possible for a hand grip, achieving a position on the wall before returning to the starting position (S6 Fig). The side-bridge (S7 Fig) and prone-bridge (S8 Fig) have been described in detail elsewhere [27].

The progression of the training programs was achieved by increasing the number of repetitions, sets, steepness of the wall, decreasing the base of support, increasing the level of the arm(s) and increasing the distance to the foot stance or hand grip. The exercises in ICT were identical, but instead of moving dynamically from a starting to a finishing position as the DCT did, the ICT performed an isometric contraction in the finishing position and maintained the position for three to five seconds, with three seconds recovery between each repetition. If a participant managed to perform the final set with the maximum number of repetitions (DCT) or for the maximum time (ICT), they progressed to a higher level at their next training session. The details of the different intensity levels are presented in Table 2 and Table 3. Numbers of climbing sessions, climbing training hours and core-training sessions were reported weekly using a login form.

**Table 2:** An overview of the progression of the training for the ICT group.

		Foot-lift*	Arm lock-off*	Prone-bridge	Side-bridge*
Level 1	Time x reps x sets Instructions	5sec x 3 x 4 Lift knee	5sec x 3 x 4 Vertical wall	15 – 20sec x 3 On the knees	15 – 20sec x 3 On the elbows
Level 2	Time x reps x sets Instructions	5sec x 5 x 4	5sec x 5 x 4	15 – 20sec x 3 ↑ distance	25 – 30sec x 3
Level 3	Time x reps x sets Instructions	5sec x 3 x 4 Lifting foot, > 90° hip angle	5sec x 3 x 4 ↑ steeper wall	25 – 30sec x 3 ↑ distance	15 – 20sec x 3 ↑ distance
Level 4	Time x reps x sets Instructions	5sec x 5 x 4	5sec x 5 x 4	15 – 20sec x 3 On the toes	25 – 30sec x 3
Level 5	Time x reps x sets Instructions	5sec x 3 x 4 Lifting hip, < 135° hip angle	5sec x 3 x 4 ↑ distance to hand grip	15 – 20sec x 3 ↑ distance	15 – 20sec x 3 Extended arm
Level 6	Time x reps x sets Instructions	5sec x 5 x 4	5sec x 5 x 4 ↑ steeper wall	25 – 30sec x 4	15 – 20sec x 3 Elevated the foot
Level 7	Time x reps x sets Instructions	5sec x 3 x 4 ↑ distance to foot stance	5sec x 5 x 4 ↑ distance to hand grip	25 – 30sec x 4 Elevated one foot	25 – 30sec x 3

* = the exercise was performed with the right and left arm or foot

**Table 3:** An overview of the progression of the training for the DCT group.

		Foot-lift*	Arm lock-off*	Prone-bridge	Side-bridge*
Level 1	Reps x sets Instructions	5–6 x 4 Lift knee	5–6 x 4 Vertical wall	5–6 x 3 On the knees	5–6 x 3 On the elbows
Level 2	Reps x sets Instructions	8–10 x 4	8–10 x 4	5–6 x 3 ↑ distance	8–10 x 3
Level 3	Reps x sets Instructions	5–6 x 4 Lifting foot, > 90° hip angle	5–6 x 4 ↑ steeper wall	8–10 x 3 ↑ distance	5–6 x 3 ↑ distance
Level 4	Reps x sets Instructions	8–10 x 4	8–10 x 4	5–6 x 3 On the toes	8–10 x 3
Level 5	Reps x sets Instructions	5–6 x 4 Lifting hip, < 135° hip angle	5–6 x 4 ↑ distance to hand grip	5–6 x 3 ↑ distance	5–6 x 3 Extended arm
Level 6	Reps x sets Instructions	8–10 x 4	8–10 x 4 ↑ steeper wall	8–10 x 4	5–6 x 3 Lifting the foot
Level 7	Reps x sets Instructions	5–6 x 4 ↑ distance to foot stance	8–10 x 4 ↑ distance to hand grip	8–10 x 4 Lifting one foot	8–10 x 4

* = the exercise was performed with the right and left arm or foot

### Statistics

To assess differences in performance, we used a two-way (groups x time) within-between analysis of variance (ANOVA) with repeated measures. When a significant interaction was detected by ANOVA, paired t-tests with Bonferroni post-hoc correction were applied to locate where the differences lay. Tests were analysed using the SPSS (SPSS 23; SPSS Inc., Chicago, IL USA) statistical software package and were analysed per protocol. The significance level was set at *p*≤0.05; all results are presented as mean±standard derivation. To quantify the strength of the findings, Cohen`s d effect size (ES) was used. An ES of 0.2 was considered a small effect, 0.5 a medium effect and 0.8 a large effect [28].

## Results

### Body lock-off

There was no difference between the groups at pre- (*p* = 0.931) or post-test (*p* = 0.592). The ICT group demonstrated a non-significant 10.6% improvement duration of the test (*p* = 0.192, ES = 0.23) while the DCT had a -0.5% reduction (*p* = 0.943) between pre-and post-testing (S9 Fig). None of the groups demonstrated greater improvement than the other group (*p* = 0.817).

### Body-lift

There was no difference between the groups at pre- (*p* = 0.372) or post-test (*p* = 0.328) in numbers of successful lifts. The ICT improved by 29.6% (*p* = 0.037, ES = 0.30) and DCT by 33.8% (*p* = 0.100, ES = 0.43), however the increases were not significantly greater than the other group (*p* = 0.333; S9 Fig).

### Superman

There was no difference between the groups at pre- (*p* = 0.561) or posttest (*p* = 0.824). A 2.4% and 5.0% improvement was observed for the ICT group (*p* = 0.248, ES = 0.19) and DCT group (*p* = 0.041, ES = 0.68) (S9 Fig). None of the groups demonstrated greater improvement than the other group (*p* = 0.457).

### MVCs of the core

No differences between the groups were observed at pre- or post-test in abdominal flexion (*p* = 0.388–0.636), rotation right (*p* = 0.555–0.782) or rotation left (*p* = 0.575–0.712, S9 Fig). In abdominal flexion, the ICT and DCT had a 10.8% and 8.7% improvement (ES = 0.50–0.51, *p* = 0.029–0.045), but neither of the groups had greater improvement than the other (*p* = 0.694). For core rotation right in DCT, there was an 8.2% improvement (*p* = 0.012; ES = 0.92) compared with 5.3% (*p* = 0.101; ES = 0.21) in ICT whereas in rotation left in DCT the improvement was 14.9% (*p* = 0.043, ES = 0.47) compared with 7.4% *p* = 0.343, ES = 0.31) in the ICT group.

### Finger hang test

Test performance improved in both groups by 4.5% ± 16.8 in DCT and 4.1% ± 20.0 in ICT; however the improvement was not significant (DCT: *p* = 0.488, ICT: *p* = 0.508). No difference in test score after the intervention resulted between groups (*p* = 0.897).

Regarding attendance, mean number of core-training sessions attended in each group was 24 ± 5 (ICT) and 25 ± 2 (DCT). In addition, 45 ± 21 (75.1 ± 39.3 hours) and 34 ± 11 (54.4 ± 25.1 hours) climbing sessions were logged (ICT and DCT; respectively) during the intervention.

## Discussion

The DCT group demonstrated improvement in three isometric core tests (trunk flexion, rotation to right and left) and in the dynamic core-strength, however, there was no improvement in two climbing-specific core tests. In comparison, the ICT group improved in isometric trunk flexion strength testing and a climbing-specific test (body-lift), involving dynamic trunk and hip activation, with no improvement in the trunk rotation or body lock-off tests. These results are not in accordance with proposals of training-specific adaptations, as the dynamically trained group in this study performed superiorly on isometric strength testing after the intervention, with some evidence of isometrically trained participants performing better on a task with dynamic trunk and hip actions.

We hypothesized there would be improvements for both groups in the climbing-specific tests: dynamic training using the exercises chosen (foot-lift and arm lock-off) requires unilateral hip flexion, either whilst the upper body supports the trunk (foot-lift) or during cross-lateral stabilization (for example, from right arm and left leg support), as the unsupported upper limb and foot reach for a hold (arm lock-off). Both exercises demand isometric trunk stiffness to provide a base of support for the limb actions, with dynamic motion produced either at the shoulder or pelvic girdle. At the volume prescribed (see Tables 1 & 2), the dose over ten weeks should have been sufficient to induce adaptation, in accordance with [29]. According to our hypothesis this protocol would be expected to increase bilateral trunk strength (as both limbs are used during training), with training in contra-lateral force transfer (opposing arm and foot reciprocally stabilized in closed chain and then engaged in open chain actions) speculated to improve transverse plane (rotational) strength, due to synergy in external and internal oblique recruitment during successful arm lock-off. In the DCT group, the outcomes were consistent with this hypothesis, with improvement recorded in isolated strength testing (sagittal and oblique biased MVC strength tests). However, the strength gains did not translate into improvement in performance of climbing-specific tests in DCT, with increase in score resulting only in the ICT trained group (significant improvement in the body-lift test). This latter finding is surprising: the body-lift test involves dynamic actions of the hip and lower spine which, according to the specificity of the training [30], should favor the DCT group, whose training included dynamic pelvis on thorax movement (bouldering arm lock-off), which has been demonstrated to evoke high EMG amplitudes in oblique abdominal muscle activation [31]. The DCT demonstrated ~34% improvement with a larger effect size 0.43 vs. 0.30 than the ICT group, which improved by 30%, however, small sample size and a large standard deviation probably resulted in a type II error for the DCT group. In addition, the body-lift test does not target the core muscles in isolation, as their actions are integrated during the test. It may be speculated that the shoulder girdle plays a significant role in assisting trunk position as the body transitions from a vertical (axial) alignment to gravity to a horizontal position, with loading of epiaxial appendicular muscles and scapulothoracic muscles. Anatomically there is contiguity and fascial interdigitation between serratus anterior and the external oblique abdominal muscles such that when the shoulder is loaded there is tensioning of surrounding myofascial.

In the other climbing specific test (body-lock), neither group demonstrated significant improvement, which is perhaps less surprising as this test is performed horizontally, a position which occurs rarely in climbing [30]. The improvements in both groups were only ⅓ of the improvements for the body-lift test. Nevertheless the test was designed to examine the ability to generate force through the entire kinetic chain, from the toes through the core and ending in the fingers [32], This is a typical pattern in climbing and is crucial for the climbers to be able to reach a new hand- or foot grip [3]. Results suggest that both approaches (ICT and DCT) had a similar impact on test result inducing a comparable magnitude of improvement in holding time, with neither group performing superiorly to the other in the post-test (mean time in test position 30 second both groups).

To our knowledge, no previous studies have compared the effect of isolated vs dynamic core training. Some, but not all previous studies have demonstrated improved performance after improved core strength and/or stability [19, 20, 22, 33]. However, there are several limitation of these studies such not using elite athletes, combining core training with general resistance training or training core stability and not core strength [10, 19, 34, 35]. Several studies have performed either dynamic, isometric interventions or combined them to examine the effects on performance [10, 11, 13, 18, 34], however as none of the abovementioned studies successfully isolated the core training modes to compare them, they are not directly comparable with the present study. Other studies have used performance measuring tests after attending a core training program, with inconclusive results: some have demonstrated no effect [11, 34] and others have demonstrated improvement in performance [13, 18, 19]. In studies which have not demonstrated an effect it has been argued that as participants were already at a high performance level, the capacity to demonstrate further improvements in performance after 8–10 weeks was minimal [11, 22, 33]. Importantly, in studies that demonstrated a positive effect [13, 18, 19], a progressive core- strengthening program was used, as has been adopted in the present study. For example Saeterbakken and colleagues showed a 4.9% improvement in throwing velocity using four levels of intensity in a core-training program, using dynamic exercises which included core stability and core strengthening [13], however as exercises were combined and not isolated into dynamic versus isometric training, as in this study, results are not directly comparable.

Core strength was measured in isolation with a dynamic test (Superman) and three isometric tests (abdominal flexion and rotation to both sides). Neither group demonstrated greater improvement than the other. However, the DCT group performed better in all tests after the intervention, while the ICT group only improved in Superman and trunk flexion. It is possible that differences in exercise performance intensity (dynamic versus isometric) could have influenced results, however, total exercise duration was matched according to previous approaches [36]. Furthermore, the improvement was between ~8–15% which is quite impressive based on baseline MVC values (94–99% of body weight) in trunk flexion pre-testing. These values are 30–50% greater than those observed in our laboratory using a comparable age-match population. Based on the pre-test results, one might conclude that climbers at a high level have a strong core as a result of climbing.

In contrast to the principle of training-specificity [30] and task-specificity [37], the isometric core-training group did not improve performance in the rotational isometric core strength tests, whereas the dynamic core training group did, which suggests the two training approaches stimulated different adaptions in soft tissue. This speculation is supported by recent evidence of mode specific training adaptations in hamstring fascicle length and muscle architecture [38, 39] and previous evidence of connecting tissue adaptations after isometric training [29].

The study has some limitations: only eighteen climbers completed the study (nine participants in each group), which increases the risk of a type II error due to low statistical power. Also as core-training was not performed in isolation, but included within participants’ overall fitness routine (i.e. climbing and bouldering), the impact of non-specific trunk loading as a result of other training activities may have had an effect on results [16]. However it is important to note that there was no difference in training status, finger hang time or climbing level between the ICT and DCT groups at baseline. Finally, as participants in this study were highly trained, these results may not be generalizable to climbers at a less elite level of participation and it may be speculated that novice or intermediate-level climbers may demonstrate greater improvements after specific core-training, as previous studies suggest [11, 12].

This study is the first of it is kind to compare the effects of dynamic and isometric core training in elite climbers and to examine whether these modes of core-training improve climbing performance. Although neither training approach demonstrated significantly greater improvement overall, the DCT improved in both sagittal and rotational core strength tests, whereas the ICT only improved in the sagittal test. Both modes of training appear equally effective in improving sagittal MVC. With regard to climbing-specific tests, only the ICT group demonstrated an improvement after the intervention, however this finding should be interpreted with caution, as a positive association between improvement in core strength and climbing performance may be harder to detect in highly trained climbers with high core strength at baseline. In summary, based on findings from this and previous comparable studies [10, 11, 13], the authors recommend that dynamic core-training should be included in climbers’ exercise regime to increase oblique muscle strength, with isometric core training adopted to maximize functional transfer of core strength gains. More research is needed to provide conclusive evidence of the transferability of improvements in core strength to performance, and to demonstrate whether dynamic or isometric protocols deliver superior performance gains.

## Supporting information

S1 Fig The Body lock-off test. (TIF)

S2 Fig The Body-lift test demonstrating the grip and the movement. (TIF)

S3 Fig The Superman test with the starting position, the pads used to measure the movement and the movement. (TIF)

S4 Fig The isometric core strength tests performed sagittaly (trunk flexion) and with rotation to both sides. (TIF)

S5 Fig The foot-lift exercise with the starting position, the movement and the finishing position. (TIF)

S6 Fig The arm lock-off exercise with the starting position, the movement and the finishing position. (TIF)

S7 Fig The side-bridge exercise with the different level of progression. (TIF)

S8 Fig The prone-bridge exercise with the different level of progression. (TIF)

S9 Fig The pre-and post results in the Body lock-off test, the Body-lift test, the Superman test and the core MVC tests. * Significant difference between pre-and post results, *p*<0.05.
(TIF)

S1 File Data set. (XLSX)
