# Supplementary figures and images for: Effects of ten weeks dynamic or isometric core training on climbing performance among highly trained climbers
Source: PLoS One. 2018 Oct 10;13(10):e0203766. doi: 10.1371/journal.pone.0203766 (PMC6179192; doi:10.1371/journal.pone.0203766)

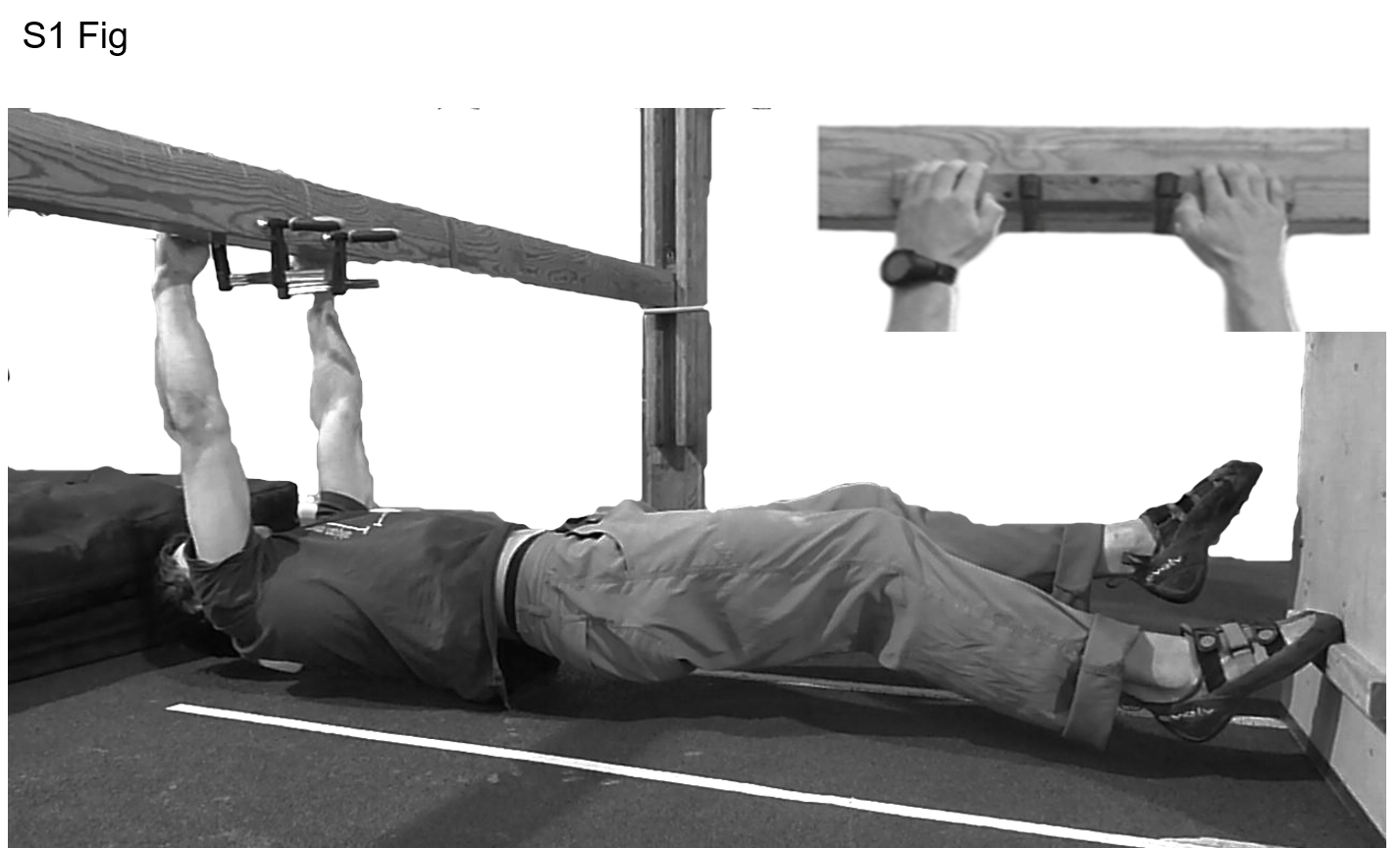

Supplement: S1 Fig — (TIF) [file pone.0203766.s001.tif]

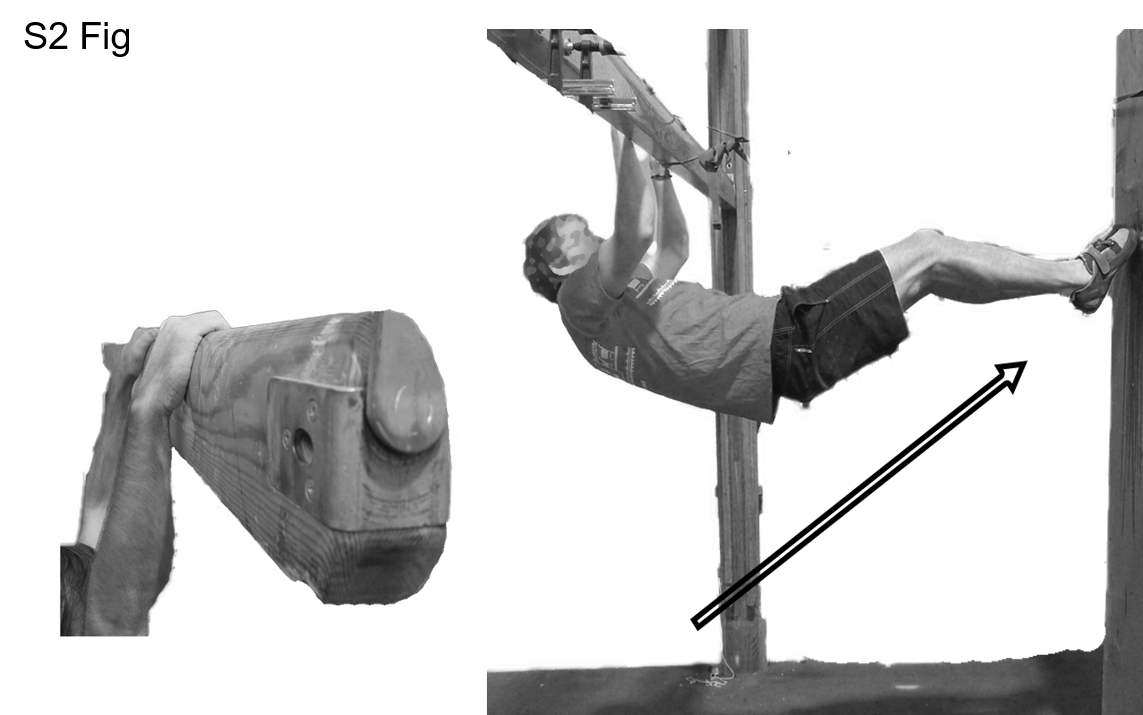

Supplement: S2 Fig — (TIF) [file pone.0203766.s002.tif]

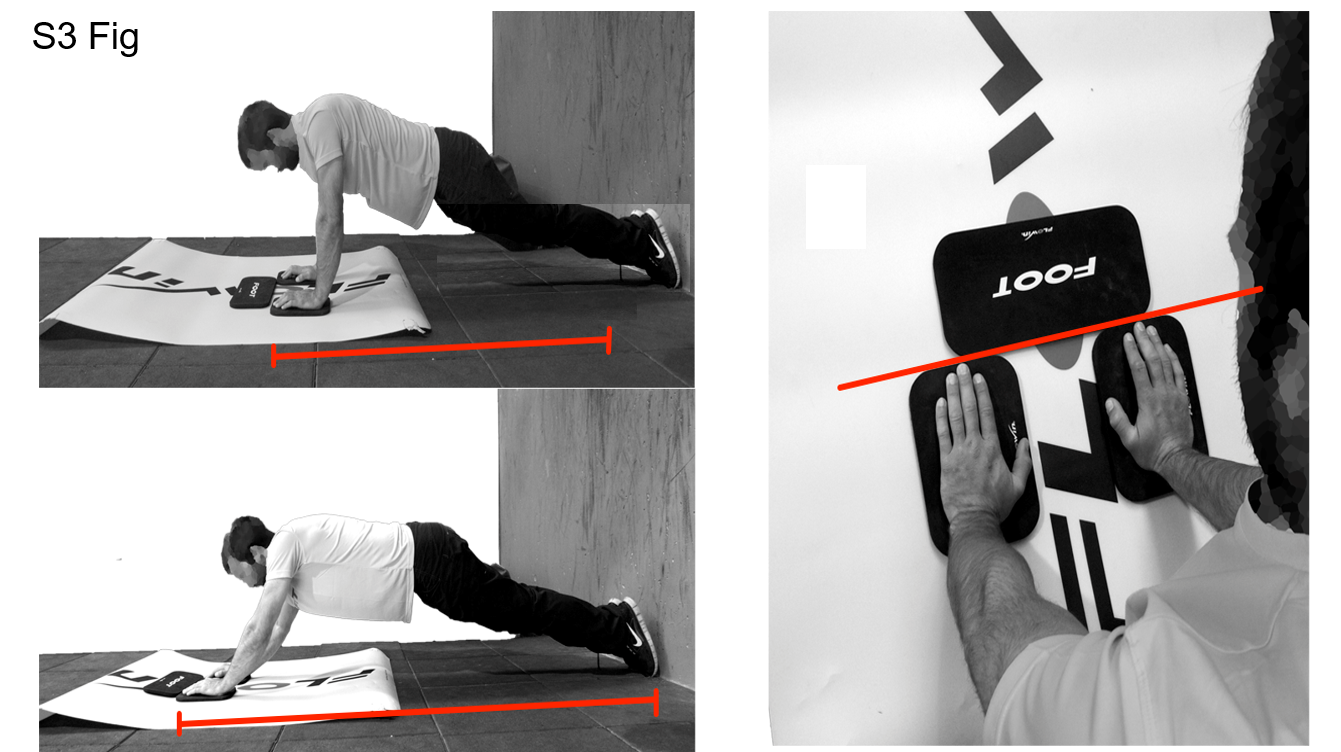

Supplement: S3 Fig — (TIF) [file pone.0203766.s003.tif]

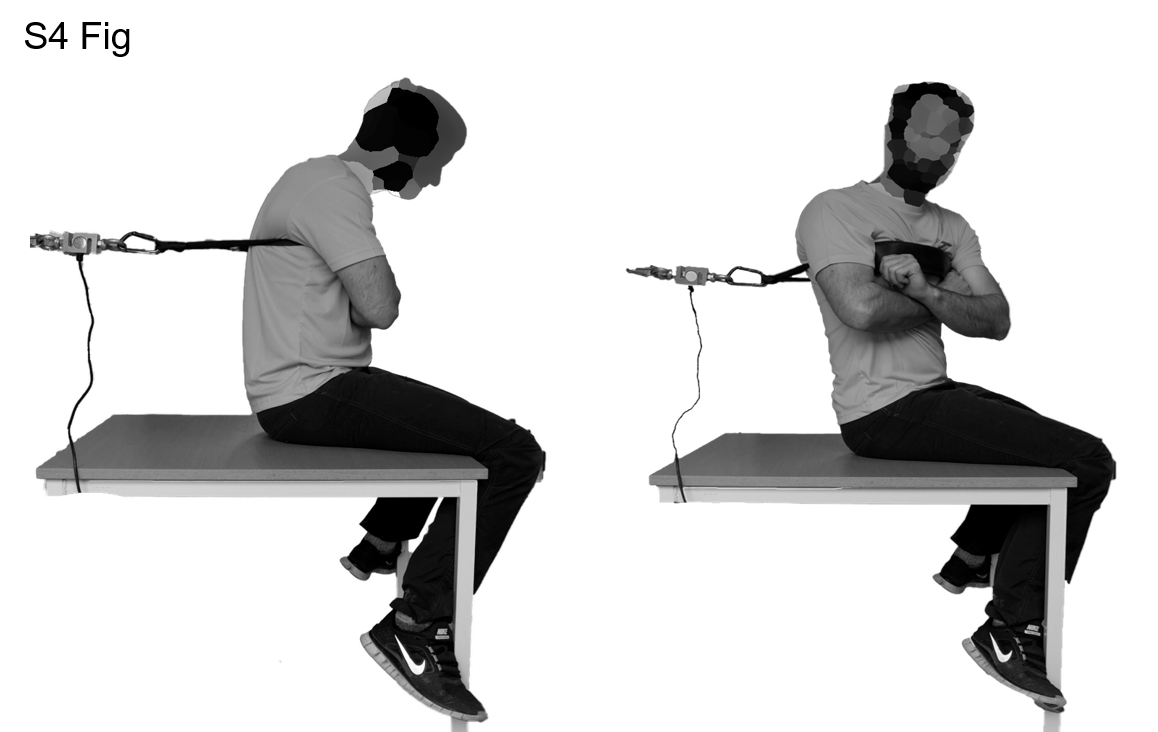

Supplement: S4 Fig — (TIF) [file pone.0203766.s004.tif]

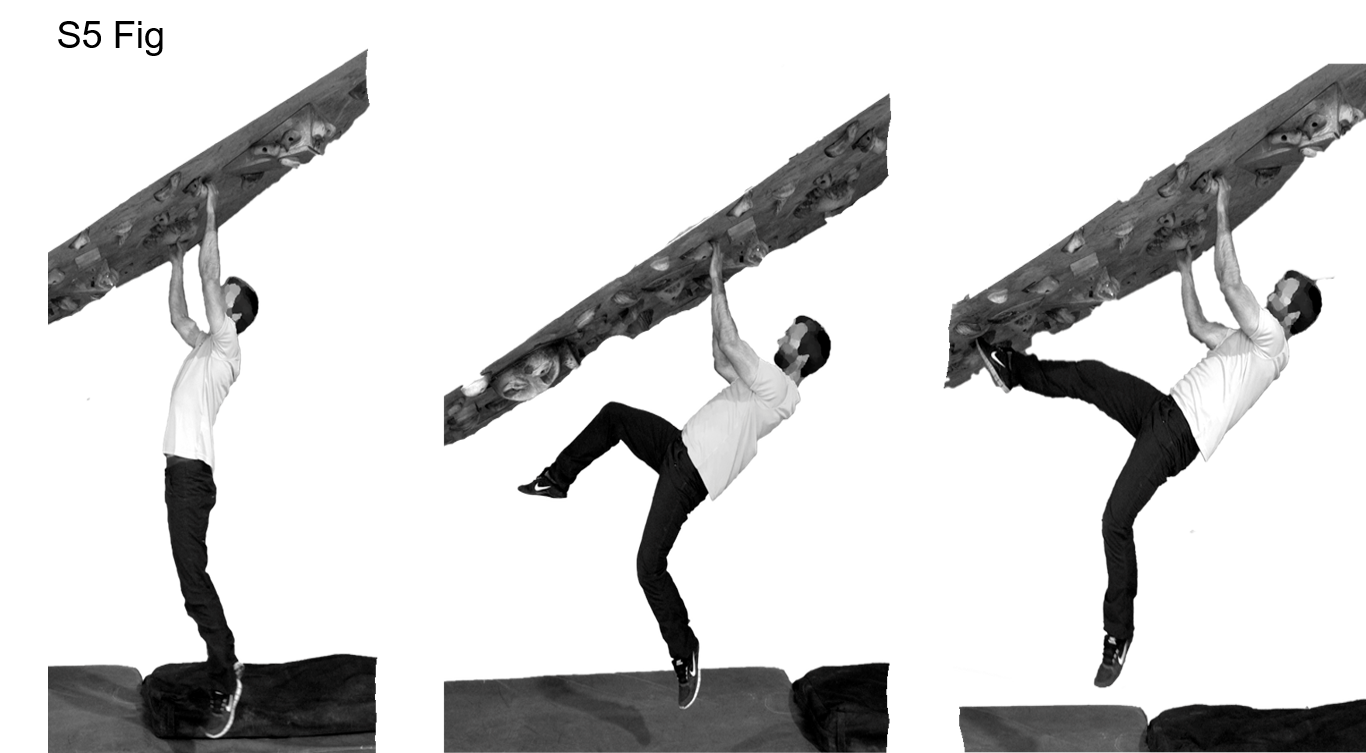

Supplement: S5 Fig — (TIF) [file pone.0203766.s005.tif]

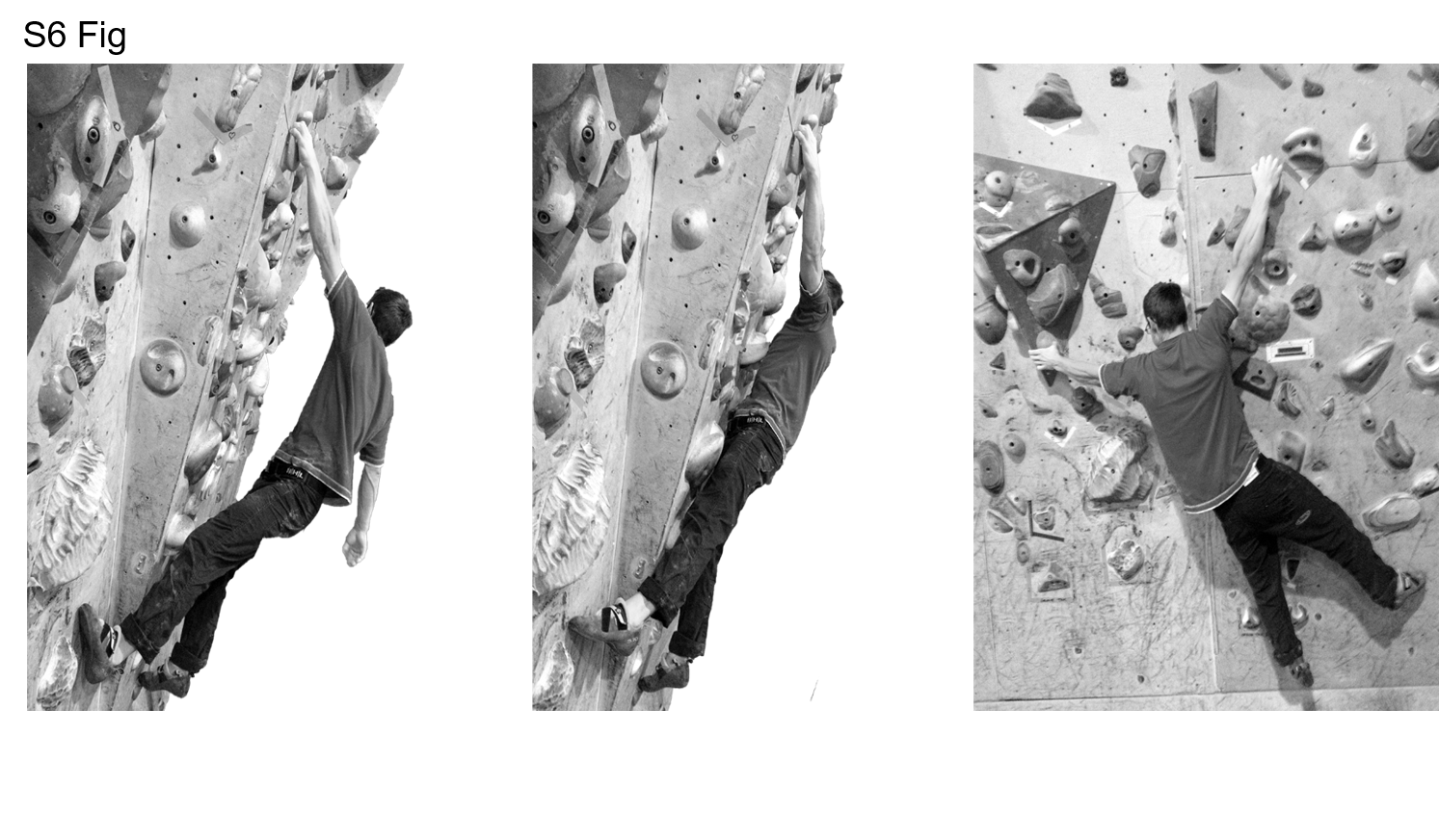

Supplement: S6 Fig — (TIF) [file pone.0203766.s006.tif]

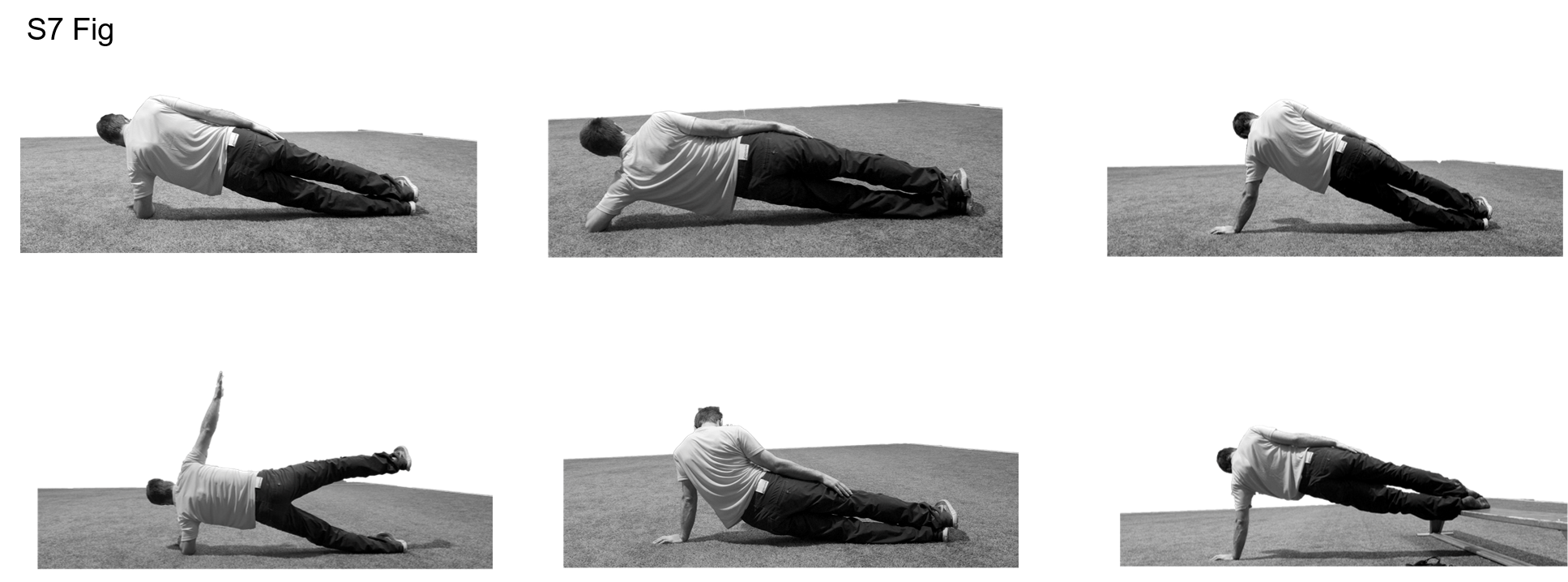

Supplement: S7 Fig — (TIF) [file pone.0203766.s007.tif]

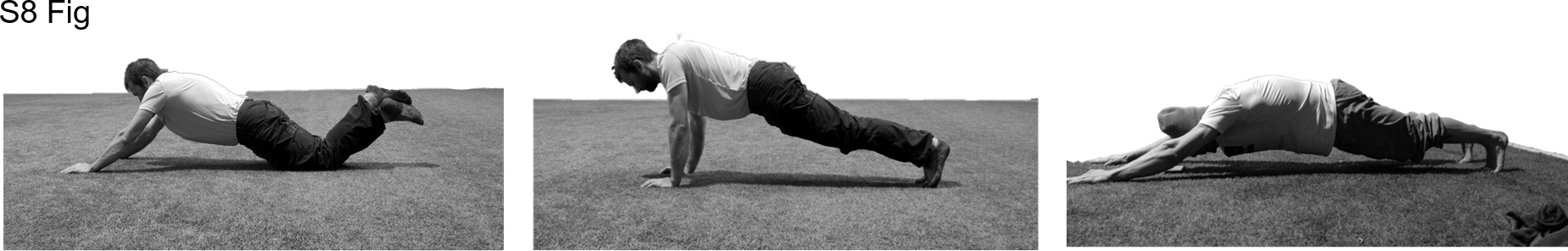

Supplement: S8 Fig — (TIF) [file pone.0203766.s008.tif]

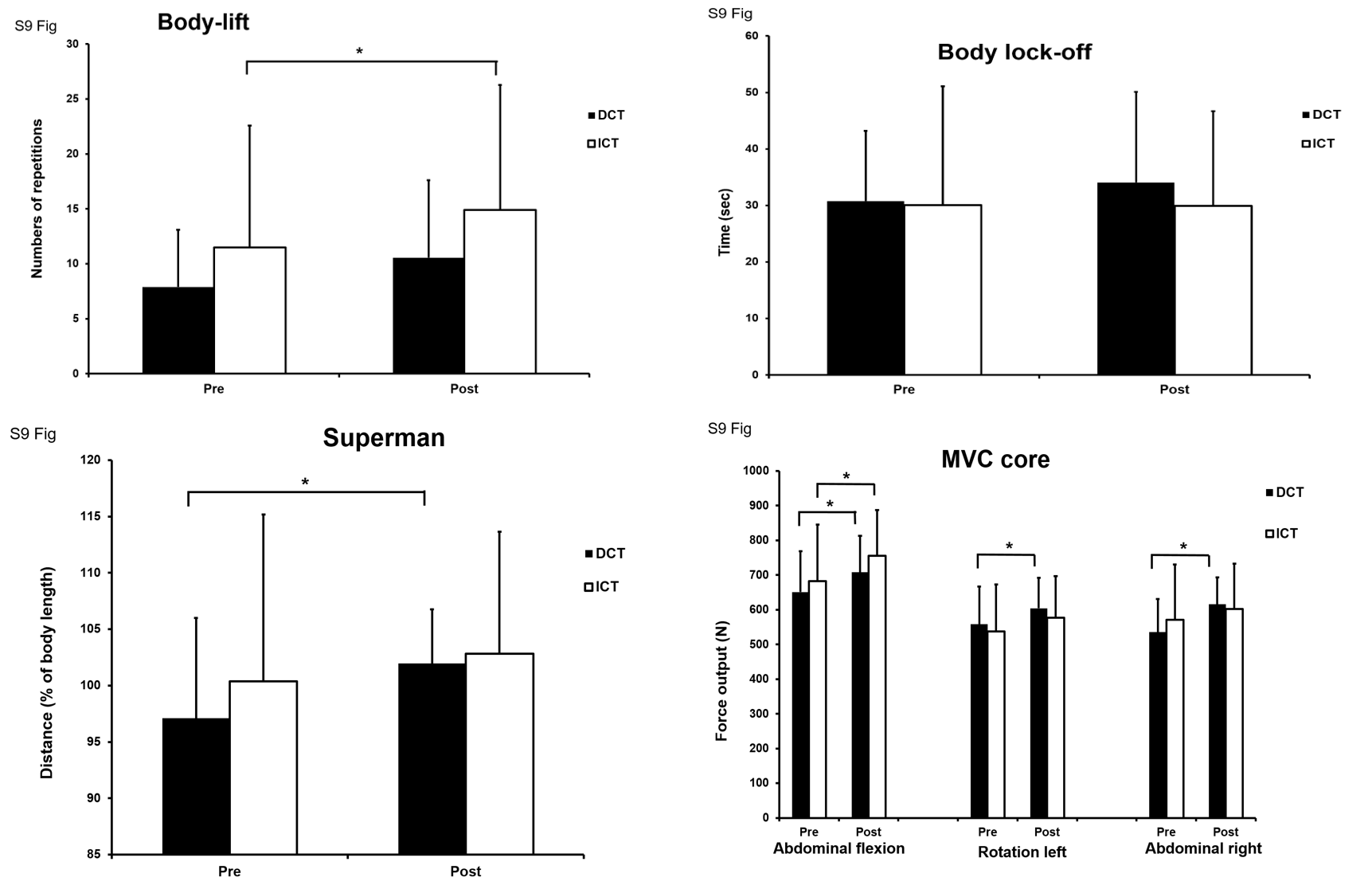

Supplement: S9 Fig — * Significant difference between pre-and post results, p<0.05. (TIF) [file pone.0203766.s009.tif]
